# Enhancing Deep Sleep Induction Through a Wireless In-Ear EEG Device Delivering Binaural Beats and ASMR: A Proof-of-Concept Study

**DOI:** 10.3390/s24237471

**Published:** 2024-11-22

**Authors:** Elke Hestermann, Kristiaan Schreve, David Vandenheever

**Affiliations:** 1 Multi-Modality Medical Imaging (M3i) Group, Faculty of Science and Technology, Technical Medical Centre, University of Twente, P.O. Box 217, 7500 AE Enschede, The Netherlands; 2 Department of Mechanical and Mechatronics Engineering, University of Stellenbosch, Joubert Street, Stellenbosch 7602, South Africa; kschreve@sun.ac.za (K.S.); davidvdh@abe.msstate.edu (D.V.); 3 Neural Engineering Research Division, Mississippi State University, 75 B. S. Hood Rd, Mississippi State, MS 39762, USA

**Keywords:** ASMR, binaural beats, delta activity, EEG, in-ear EEG, sleep staging

## Abstract

This study presents the development of a wireless in-ear EEG device designed to monitor brain activity during sleep and deliver auditory stimuli aimed at enhancing deep sleep. The device records EEG signals and plays a combined auditory stimulus consisting of autonomous sensory meridian response (ASMR) and 3 Hz binaural beats at a 60:30 dB ratio, intended to promote delta wave activity and non-rapid eye movement (NREM) stage 3 sleep. Fifteen participants completed this study, which included two consecutive nights: a baseline night and a testing night. Participants were divided into an experimental group, which received the combined ASMR and binaural beat stimulus, and a control group, which received only ASMR. The combined stimulus was delivered upon entering NREM stage 2 and replaced by ASMR when NREM stage 3 was reached. Results showed that the experimental group experienced an increase in NREM 3 sleep, a decrease in NREM 2 sleep, and a slight increase in NREM 3 latency compared to the baseline night. Although the findings are promising, further testing with a larger sample size is required to confirm the device’s potential to enhance sleep quality and promote delta activity in the brain.

## 1. Introduction

Sleep is essential for maintaining physical and mental well-being. Adults aged 18 to 64 are recommended to obtain between seven to nine hours of sleep per night [1]. However, approximately 25% of adults report that they consistently fail to achieve these recommended hours of sleep [2], leading to a variety of negative health consequences. Chronic sleep deficiency has been linked to long-term health conditions such as obesity, type 2 diabetes, and cardiovascular disease, underscoring the importance of both sleep duration and quality [3].

Sleep quality is closely tied to brain activity, particularly within specific frequency ranges. According to Abeln et al., the quality of sleep can be assessed by examining the amount and amplitude of three key frequency bands: delta, theta, and alpha [4]. These bands are especially prominent during different stages of sleep, with deep sleep (NREM3) being characterized by increased delta wave activity.

The definition of sleep quality has been vaguely defined in research until Nelson et al. [5] clarified its meaning using Rodgers’ Evolutionary method to identify key attributes. They outlined four core components of sleep quality: sleep efficiency, sleep latency, sleep duration, and wake after sleep onset. Additionally, Shrivastava et al. [6] defined sleep efficiency in 2014 as the proportion of time spent asleep in bed relative to the total time spent in bed, encompassing all sleep stages (NREM 1, NREM 2, NREM 3, and REM). Shrivastava et al. [6] also defined sleep latency as the time taken to reach the first sleep stage, while NREM 3 latency is the time from sleep onset to the first NREM 3 epoch. Given that NREM 3 is predominantly present during the first third of the night (around 20%), it is crucial to have adequate amounts of NREM 3 sleep for restorative processes [7]. Electroencephalogram (EEG) technology offers a reliable method to measure these brain wave frequencies and identify which stage of sleep the brain is currently in. Knowing the brain wave frequencies and sleep stages, it is possible to determine participant’s sleep efficiency and NREM 3 latency.

The current gold standard for sleep monitoring is polysomnography (PSG), a comprehensive method that utilizes EEG alongside electro-oculogram (EOG) and electromyogram (EMG) recordings to assess sleep stages. However, PSG is both resource-intensive and intrusive, as it requires individuals to sleep in a laboratory setting connected to numerous wires, often leading to discomfort and poor sleep quality. Given the limitations of PSG for routine at-home use, there is a growing need for more practical, non-intrusive sleep monitoring devices that can deliver high-quality data.

One promising alternative is the use of in-ear EEG technology, which was first introduced by Looney et al. [8]. This technology utilizes electrodes placed inside the ear canal to measure brain activity, offering several advantages over traditional scalp EEG. In-ear EEG devices are more discreet, user-friendly, and less prone to motion artifacts, making them a viable option for sleep monitoring in real-world environments [9,10]. The constant pressure of the earbud against the skin provides stable recordings without the need for expert placement, unlike the complex setup required for scalp EEG [9,10].

In addition to sleep monitoring, in-ear EEG devices have the potential to deliver interventions aimed at improving sleep quality. Auditory stimuli, such as music, have shown promise in influencing brain activity to promote deeper sleep [11,12]. Another promising auditory stimulus is binaural beats, which have garnered attention for their potential to enhance sleep quality. Binaural beats are an auditory illusion created when two slightly different frequencies are presented to each ear. This causes the brain to perceive a third, rhythmic frequency corresponding to the difference between the two tones [13,14,15,16]. Studies have demonstrated that binaural beats, especially those in the delta frequency range, can increase the duration of NREM3 sleep, the deepest stage of non-rapid eye movement (NREM) sleep [17,18].

A key challenge with binaural beats, however, is that some individuals find the repeated auditory stimulus uncomfortable or anxiety-inducing [19]. To mitigate this, it is proposed to combine binaural beats with autonomous sensory meridian response (ASMR), a stimulus known to promote relaxation and calmness [20]. ASMR, often described as a tingling sensation triggered by specific auditory or visual stimuli, has been used successfully in previous studies to reduce anxiety and promote sleep [20].

This study aims to design and evaluate an in-ear EEG device that not only monitors sleep stages but also uses a combined auditory stimulus of ASMR and a 3 Hz binaural beat to promote deeper sleep, specifically targeting the NREM3 stage. By combining these auditory stimuli and utilizing a non-intrusive in-ear EEG system, this device has the potential to offer a novel, user-friendly solution for improving sleep quality in natural home environments.

## 2. Materials and Methods

### 2.1. Earpiece Design

**Figure 1:**
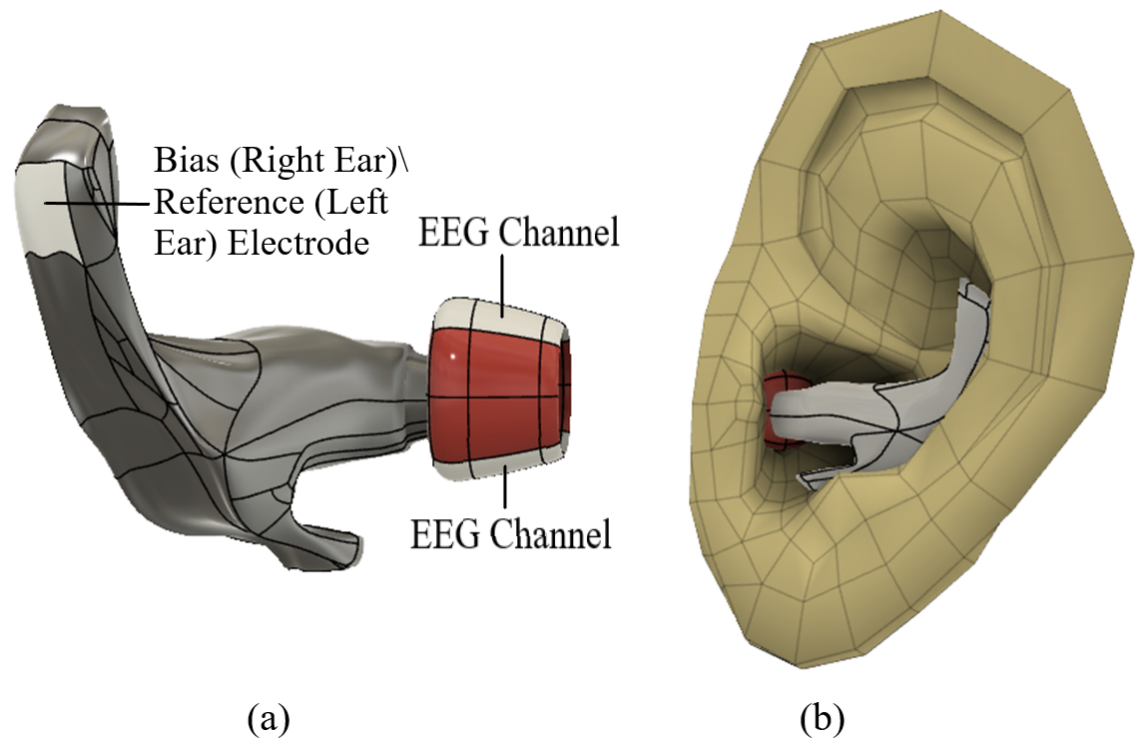
Design of earpiece. Part (**a**) illustrates the earpiece where the section in red represents the part inserted inside the ear canal, and the light gray shows the placement of the in-ear EEG, bias and reference electrodes depending on the ear. Part (**b**) demonstrates how the earpiece would be inserted in the ear.

The in-ear EEG device includes an earpiece with two electrodes positioned inside the ear canal and an additional electrode on the concha for each ear, based on the design adapted from [21]. The final concept design, created in the software program Autodesk Fusion Version 16.0 [22], can be seen in Figure 1. The earpiece features stabilizing wings that maintain secure contact with the concha, minimizing the risk of dislodgement during sleep.

**Figure 2:**
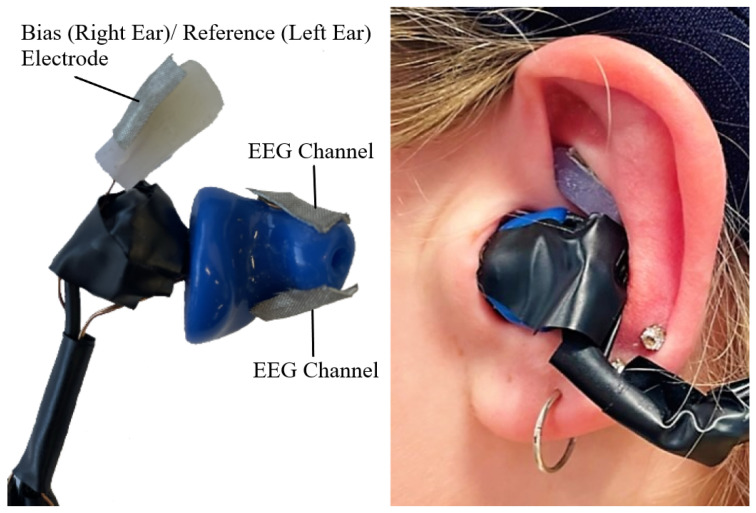
The earpiece design with a close-up of the earpiece on the (**left**) and how it fits in the ear on the (**right**).

The final design prototype is shown in Figure 2. Electrodes consisted of enamel wire soldered to 5 mm × 10 mm copper tape covered with conductive cloth, selected for its low resistance, flexibility, and comfort, as validated by prior studies [23,24,25]. Double-sided conductive tape attached the electrodes to the earpiece, with reference and ground electrodes placed at the concha cymba [26]. Exposed wires were shielded for participant safety. The earpiece structure was molded from medical-grade silicone, with the 7 mm earbud extending no further than 10 mm into the ear canal, following the guidelines of [10].

### 2.2. EEG Processing Board

The EEG processing board utilized in this study is OpenBCI’s Cyton board (OpenBCI, Brooklyn, NY, USA) [27]. Although the Cyton board supports eight channels, only four were used here. The Cyton features a 24-bit ADS1299 (Texas Instruments, Texas, Dallas, TX, USA) analog-to-digital converter (ADC) [28], designed for low-noise biopotential data acquisition and adaptable to specific design requirements. With a high input impedance of 1 GΩ, the ADS1299 minimizes potential skin-contact errors [29]. Equipped with a Bluetooth dongle, the board can connect to a laptop via Bluetooth or serial link and is powered by an external battery.

**Figure 3:**
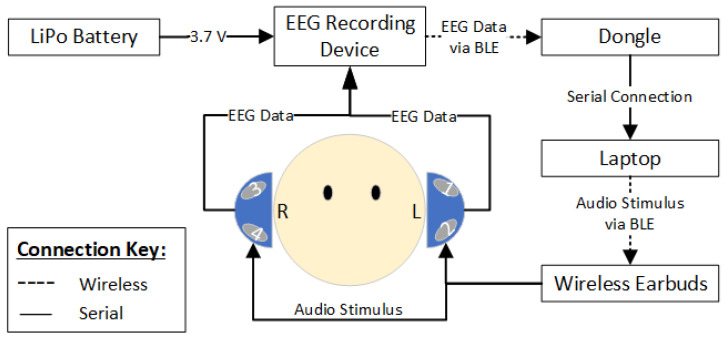
System architecture and the linkage between the devices.

In this study, EEG data were transmitted via Bluetooth from the Cyton board to a laptop positioned near the participant’s head using a dongle. Audio stimulation was delivered through Sony WI-C200 wireless Bluetooth stereo earphones (Sony Group Corporation, Tokyo, Japan) paired with the laptop. Stereo playback was essential for the audio stimulus, supported by a 3.7 V, 1000 mAh battery to sustain continuous streaming. The Cyton board, drawing 65 mA when connected, ensured consistent operation throughout testing. Figure 3 illustrates the system architecture and device connectivity.

### 2.3. Final Device

**Figure 4:**
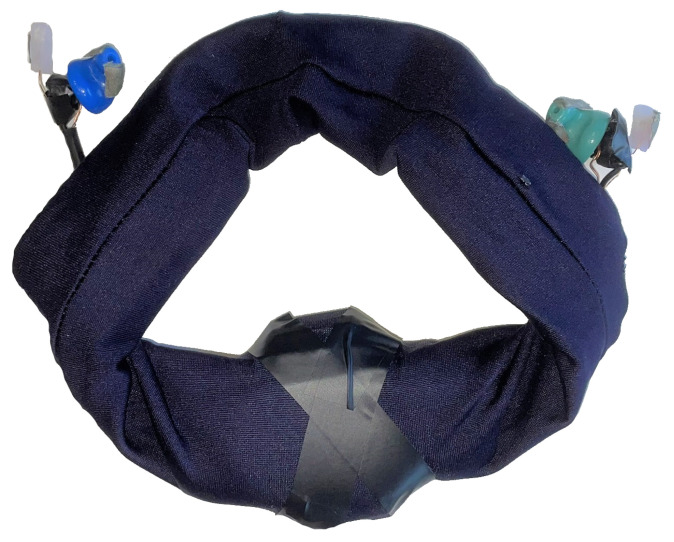
Final design of in-ear EEG device.

**Figure 5:**
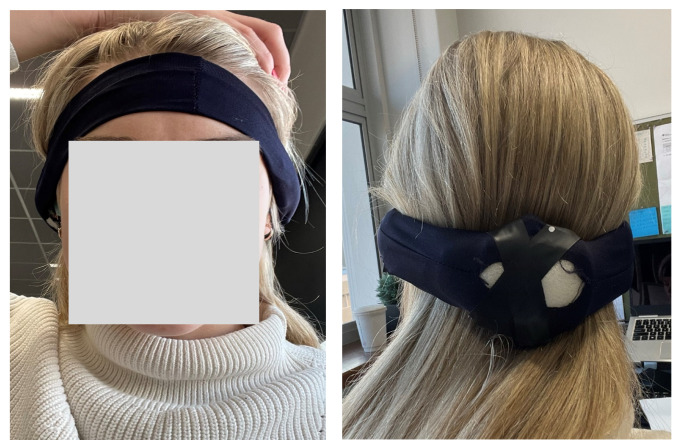
Front and back view of how the participants wore the in-ear EEG device.

The complete headpiece device comprises four components: the Cyton board for EEG recording with its own 3D-printed casing, Sony Bluetooth earphones for audio delivery, a silicone earpiece for EEG data collection, and a LiPo battery to power the Cyton board. All components are encased within a soft elastic headband for participant comfort, as shown in Figure 4. In this configuration, the dark blue earpiece serves as the left ear electrode, and the light green as the right one. When worn, the Cyton board is positioned at the base of the skull, with the headband securing the earpieces as illustrated in Figure 5.

### 2.4. Auditory Stimulus

The auditory stimulus used in this study combined ASMR with a specific binaural beat. This beat featured a 250 Hz carrier tone for the left ear and a 253 Hz tone for the right ear, creating a 3 Hz binaural beat within the delta range, ideal for NREM stage 3 sleep. To enhance comfort, a 60:30 dB ratio of ASMR to binaural beat was adopted, following [30]. The ASMR stimulus used ocean wave sounds, which are known for their calming effects. The stimuli were created using Audacity software Version 3.2 [31]. In the experimental group, the in-ear EEG device delivered the combined auditory stimulus upon detecting five consecutive NREM 2 epochs, switching to ASMR alone after five consecutive NREM 3 epochs. The decision to implement five consecutive epochs in this study was based on the standard practice utilized by a local clinical neurophysiologist at a sleep clinic for identifying sleep stages. According to this protocol, a sleep stage is only classified as being achieved when five consecutive epochs of that specific stage are observed.

### 2.5. Sleep Stage Identifiers

The recorded data were analyzed to classify five sleep stages—wakefulness, NREM 1, NREM 2, NREM 3, and REM—each associated with a distinct frequency range. Sleep staging was conducted using YASA [32], an open source, automated tool, which applies pre-trained classifiers validated on 3000 nights of data from the National Sleep Research Resource. Every 30 s, YASA assessed and predicted participants’ sleep stages. Previous validation studies by YASA’s developers have shown a median accuracy of 86.6% when compared to expert human scorers in healthy adults. [32].

This study examines key sleep parameters, including the duration of NREM stages 2 and 3 (Equation (1)), latency to NREM stage 3, sleep efficiency, and relative theta and delta band power. All parameters are presented as percentages, with the exception of theta and delta band powers. Sleep efficiency, a standard measure in insomnia treatment evaluation [33], is calculated as the ratio of total sleep time (TST) to time spent in bed (Equation (2)). TST excludes awake epochs (Equation (3)), while total bed time includes all sleep epochs (Equation (4)). Baseline and stimulation nights were assessed independently for accuracy.

(1) $$\%\mathrm{Sleep}\;\mathrm{Stage}=\frac{\mathrm{Duration}\;\mathrm{of}\;\mathrm{Sleep}\;\mathrm{Stage}}{\mathrm{Total}\;\mathrm{Sleep}\;\mathrm{Time}}$$

(2) $$\%\mathrm{Sleep}\;\mathrm{Efficiency}=\frac{\sum \mathrm{Duration}\;\mathrm{of}\;\mathrm{Sleep}\;\mathrm{Stages}}{\mathrm{Total}\;\mathrm{Time}\;\mathrm{in}\;\mathrm{Bed}}$$

(3) Total Sleep Time =∑NREM 1, NREM 2, NREM 3, REM

(4) Total Time in Bed=∑NREM 1, NREM 2, NREM 3, REM, Wake

### 2.6. EEG Data Analysis

The EEG data collected via the Cyton board was processed using Brainflow, an API from OpenBCI designed to integrate Cyton firmware with Python, enabling efficient data handling through basic Python Version 3.10 functions [34]. Data were imported and analyzed within a Jupyter Notebook Python script. The Cyton board, sampling at 250 Hz using the ADS1299 amplifier, stored raw EEG data in a .csv file for subsequent processing. The data were then reformatted into an MNE Python raw array, with the last four unused channels of the Cyton board excluded to meet the YASA automatic sleep staging function’s input requirements [35].

A 50 Hz notch filter was applied to remove power line harmonic noise from the data. This filter was implemented using a finite impulse response (FIR) approach with the Hamming windowing method, achieving a passband ripple of 0.0194 and a stopband attenuation of 53 dB. Subsequently, a fourth-order Butterworth bandpass filter was applied to reduce noise from suboptimal skin contact and movement during sleep. The filter, with a lower limit of 0.5 Hz and an upper limit of 15 Hz, effectively isolated sleep frequencies while minimizing wakefulness interference. Forward-reverse filtering was employed to eliminate phase shifts, enhancing signal accuracy.

After the MNE-Python array was filtered, it was processed through the YASA automatic sleep staging function. The data were first resampled to 100 Hz to enhance computational efficiency, a crucial factor for real-time analysis of continuous data every 30 s. Sleep stages were then determined following the resampling process. Sleep statistics were computed using the YASA library, adhering to the guidelines set by the American Academy of Sleep Medicine (AASM) [7].

Post-data collection was performed using Python [36] within the Jupyter Notebook environment [37]. Spectral analysis of the filtered EEG data were conducted via fast Fourier transform (FFT) and power spectral density (PSD) techniques. These analyses provided insights into the influence of the audio stimuli on participants, highlighting the dominant frequencies. The PSD was calculated using the Welch method, which minimized variance by averaging small overlapping segments. Total band power was computed using Simpson’s rule. Relative band power ($BP_{relative}$) for each frequency range was derived by dividing the frequency-specific band power ($BP_{frequency}$) by total band power ($BP_{total}$), as shown in Equation (5). Sleep stage percentages were calculated as the duration of each stage relative to total sleep time (TST). Sleep stage latency was calculated as the time to the first epoch of the desired stage, with longer latencies potentially indicating poorer sleep efficiency.

(5) $$BP_{relative}=\frac{BP_{frequency}}{BP_{total}}\;$$

Following data filtration, statistical analysis was performed to assess the significance of differences between the two testing nights across the baseline, experimental, and control groups. A mixed-model ANOVA was employed to evaluate the impact of the two testing nights and the effects of auditory stimuli. This approach was selected because participants served as their own control, with comparisons made between their baseline and audio stimulus nights. While a 95% confidence level was targeted, a 90% confidence level was also considered meaningful due to the small sample size. All statistical analyses were conducted using Statistica Version 14 [38].

### 2.7. EEG Channel Rejection

**Figure 6:**
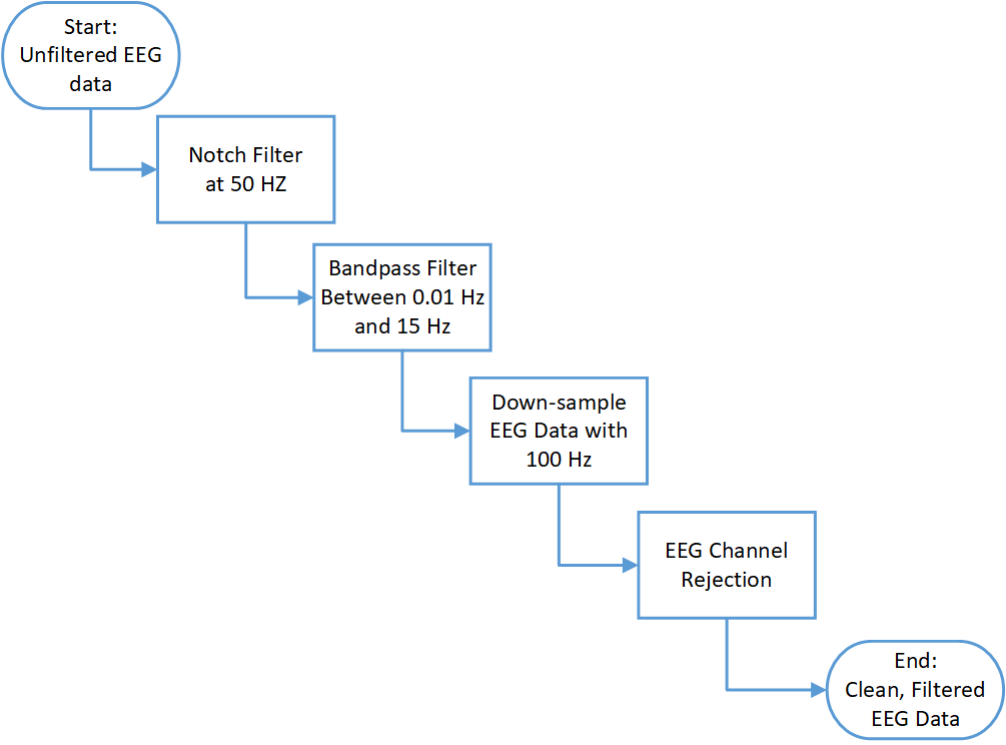
Flow diagram of the EEG data filtering procedure.

EEG data are susceptible to artifacts, which must be systematically removed to ensure the integrity of the dataset. To address this, we followed the channel rejection protocol outlined in [39]. Initial filtering was performed using a notch and bandpass filter. Any data points outside the dynamic range were replaced with ‘NaN’ and excluded from further analysis. Electrode movement within the ear caused significant amplitude spikes, which were mitigated through filtering. Channels with suboptimal skin contact, resulting in dominance of high-frequency noise, were also rejected. All rejected data and channels were replaced with ‘NaN’ and not included in subsequent analyses. Figure 6 illustrates the filtering and down-sampling process applied to the EEG data.

### 2.8. Validation Test

Prior to initiating formal testing, several validation tests were conducted to ensure the proper functionality of the in-ear EEG device. The first test aimed to verify the signal output of the conductive cloth electrodes. Since EEG signal amplitudes are typically in the microvolt range, it was essential to confirm that the conductive cloth electrodes could reliably capture signals within this range. To do so, the electrodes were connected to a signal generator, and the output was measured via an oscilloscope to ensure correlation with the input signal. The signal generator supplied a 1 kHz sine wave to the conductive cloth electrode, and it was expected that the electrode would have output a 1 kHz sine wave output on the oscilloscope. The 1 kHz sine wave signal was used to test if the constructed conductive cloth electrode could output the correct signal.

The second validation test aimed to assess whether the proposed device accurately outputs EEG sleep signals. A healthy volunteer (female, age 22) with no known health issues wore the device for 60 s and then for a brief 30 min sleep session in a familiar environment such as their home. The healthy volunteer followed the same experimental protocol which was used in this study and described below in Section 2.10. The EEG data were collected in 30 s epochs throughout 30 min. After collecting the EEG data from the healthy volunteer, the data were filtered and processed with the same methods as described above which will be used for this study. This provided a clear indication that the EEG data analysis protocol would be sufficient data for this study. The resulting EEG data were compared to data from a previous study [17] to determine if the output was consistent and realistic.

### 2.9. Participants

Fifteen individuals participated in the study, including four males and eleven females, with a mean age of 30.73 ± 11.77 years. Participants ranged from 18 to 64 years old, were healthy, and had no history of sleep or hearing disorders. They were permitted to take prescribed medications, excluding medication that could induce drowsiness. To ensure consistency, participants followed a set of guidelines:

1. Sleep duration of at least six hours;
2. Consistent bedtime across both nights;
3. No caffeine or nicotine after 3:00 PM;
4. Avoidance of alcohol, sleep aids, or drugs;
5. No exercise within two hours of bedtime.

These measures were implemented to minimize external factors affecting sleep consistency in the home environment. Participants were required to fully understand the study requirements and provide written informed consent, with the option to withdraw without explanation. All data were anonymized. This study was ethically approved by the Health Research Committee 1 (HREC 1) of the University of Stellenbosch (reference number: S20/08/223).

### 2.10. Experimental Procedure

The experimental procedure involved each participant using an in-ear EEG device for two consecutive nights: a baseline night and an auditory stimulation night. During the baseline night, participants wore the device without receiving any auditory stimulus. Participants were then randomly assigned to either a control group, receiving only ASMR, or an experimental group, receiving both ASMR and a 3 Hz binaural beat. In the experimental group, the combined stimulus began upon reaching NREM stage 2 and stopped at NREM stage 3, repeating for a 5 h EEG recording period. This audio stimulus delivery approach was adapted from [17]. Out of the 15 participants, 8 received the combined audio stimulus of ASMR and the 3 Hz binaural beat, and 7 participants received only the ASMR.

## 3. Results

**Figure 7:**
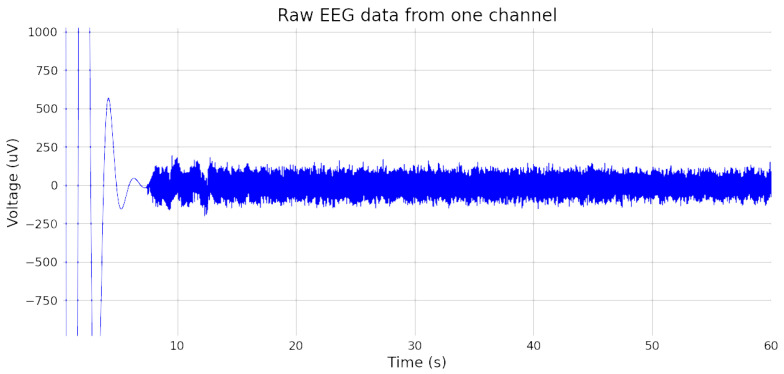
Unfiltered, raw EEG data of one channel.

The signal verification test confirmed the reliability of the selected electrodes. Figure 7 presents the 60 s recording of raw EEG data using the conductive cloth and EEG device, demonstrating the in-ear EEG system’s capability to capture low-amplitude EEG signals in the microvolt range. The EEG recording was taken in a relaxed wakefulness state with eyes closed. After 10 s, the device provided stable EEG data, validating the device’s effectiveness for accurate EEG signal acquisition.

**Figure 8:**
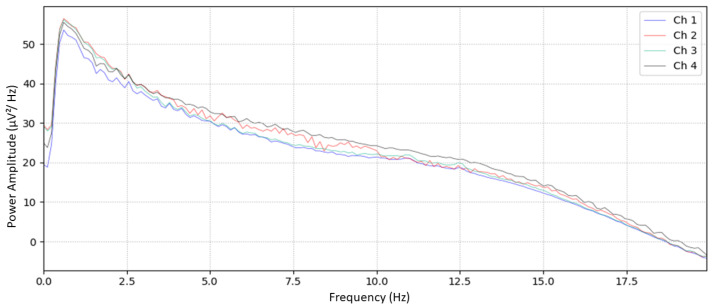
Obtained EEG sleep data from the designed in-ear EEG device.

A second validation test involved collecting sleep EEG data from a healthy volunteer using the designed device. The device’s data were compared to findings from a similar study by Jirakittayakorn and Wongsawat [17], which informed this study’s audio delivery protocol. Unlike the previous study of Jirakittayakorn and Wongsawat [17], which solely employed a 3 Hz binaural beat and recorded EEG via scalp electrodes, this study used a combined audio stimulus. Figure 8 presents the power spectral density (PSD) of the four EEG sleep channels, providing insight into the device’s comparative performance across sleep stages.

Figure 8 displays a distinct peak between 0 Hz and 2.5 Hz, indicating strong delta frequency activity. This peak declines as frequency increases, suggesting that lower frequencies dominate, affirming the device’s capability to capture low-frequency sleep signals effectively. The PSD graph of Jirakittayakorn and Wongsawat [17] (Figure 5) shows a comparable pattern of delta activity in NREM 2 sleep, with a prominent peak and subsequent decline. The close similarity between the PSD profiles from this study and [17] supports the device’s reliability in recording EEG sleep data.

### Experimental Results

**Figure 9:**
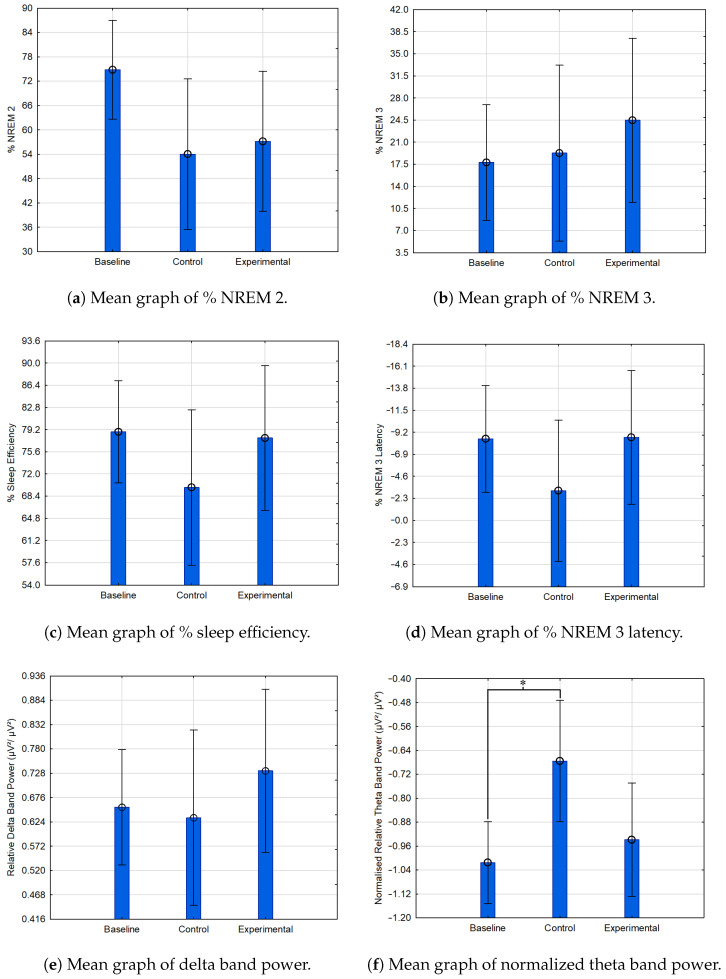
Comparison between the baseline, experimental, and control groups, where an asterisk (*) indicates statistical significance. The error bars present the standard deviation relative to the mean.

A total of 120 EEG channels were initially recorded, with 107 channels retained following necessary rejections. The two-night assessment yielded an average of 497.97 min of data, with 250.6 min for baseline and 247.37 min for testing. Baseline night data across participants were combined into a single baseline group. Figure 9 displays the mean values for each parameter. Due to an outlier in relative theta band power, data normalization via logarithmic transformation was applied, as shown in Figure 9f.

Figure 9a shows that both audio-stimulus groups exhibited reduced NREM 2 sleep relative to baseline, with the control group experiencing the least NREM 2 sleep. The experimental group demonstrated the highest percentage of NREM 3 sleep, correlating with increased delta band power (Figure 9b,e), consistent with delta frequencies’ alignment with NREM 3. Additionally, the experimental group scored higher on sleep efficiency than the control group, though slightly lower than the baseline group (Figure 9c). As seen in Figure 9d, the experimental group had the greatest NREM 3 latency, followed by the baseline and control groups.

The *p*-values were calculated using ANOVA with post hoc tests, where potential power issues were noted—likely due to the small sample size and high *p*-values observed. Despite potential power concerns, relative theta band power showed significance both pre- and post-normalization, with *p*-values of 0.04 and 0.01, respectively, for comparisons between baseline and control groups. The time spent in NREM 2 for baseline vs. control yielded a *p*-value of 0.07, approaching a 93% confidence level, while baseline vs. experimental yielded a *p*-value of 0.1 (90% confidence). NREM 3 latency for control vs. baseline and theta band power for experimental vs. control showed *p*-values of 0.13, aligning with an 87% confidence level.

## 4. Discussion

The findings from this study offer preliminary evidence for the effectiveness of in-ear EEG devices in monitoring sleep and delivering auditory stimuli. Participants in the experimental group, who received ASMR and a 3 Hz binaural beat stimulus, demonstrated the highest percentage of NREM 3 sleep (24.50%) compared to the baseline (17.80%) and control groups (19.28%). This suggests that combined auditory stimuli may promote deeper, restorative NREM 3 sleep stages [40,41]. Additionally, the experimental group exhibited the highest delta band power (0.73), indicating enhanced slow-wave activity and reinforcing the link with increased NREM 3 sleep.

The control group, which received only the ASMR stimulus, spent the least time in NREM 2 sleep (54.01%) compared to 57.16% in the experimental group and 74.80% in the baseline group. This reduction, alongside increased theta band power, aligns with prior research, suggesting that ASMR alone can promote a relaxed state associated with heightened theta activity. Additionally, the experimental group showed a higher latency to NREM 3 (8.67%) than the baseline and control groups (8.51% and 3.26%, respectively), suggesting that increased NREM 3 duration may contribute to extended latency for subsequent NREM 3 phases.

While the experimental data are promising, statistical analysis highlights limitations arising from the small sample size. The only statistically significant finding, after normalization, was the difference in theta band power between the control group and its baseline (*p* = 0.01). Other measures, including the percentage of NREM 2 between the experimental and baseline groups (*p* = 0.10) and normalized theta band power between the control and experimental groups (*p* = 0.07), approached significance. These findings underscore the need for a larger sample to enhance statistical power and assess the effects of the combined auditory stimulus more robustly.

Current sleep studies commonly use scalp EEGs with numerous wired connections, which may cause discomfort. For instance, studies by Jirakittayakorn and Wongsawat [17,18], Lee et al. [30], and Walsh et al. [42] utilized scalp EEG, while a limited number, including [39,43,44], have explored in-ear EEG. However, these in-ear devices still required wires to connect the earpieces to processing units. Additionally, audio stimulus delivery has traditionally relied on separate devices, highlighting the potential for developing a wireless in-ear EEG system that simultaneously records data and delivers auditory stimuli during sleep.

In 2018, Jirakittayakorn and Wongsawat [17] investigated the effects of a 3 Hz binaural beat on sleep stages, finding increases in delta band power, enhanced NREM 3 sleep, and reduced NREM 2—findings that align with the present study’s results. Here, the experimental group also showed greater sleep efficiency (77.83%) compared to the control, with the baseline group scoring slightly higher (78.83%), suggesting that the auditory stimulus did not disrupt participants’ sleep. While Lee et al. [30] employed a combined ASMR and 6 Hz binaural beat to induce the theta state, leading to increased theta power, this proof-of-concept study aimed to induce delta state using a 3 Hz binaural beat. Notably, both previous studies used scalp EEG, while the present study achieved comparable results with an in-ear EEG device, supporting its feasibility for EEG monitoring and auditory stimulus delivery. However, certain limitations remain.

The electrodes used in this study were made from conductive cloth, a material previously demonstrated as suitable for EEG recordings [23,24,25,43]. However, we observed some degradation in the conductivity and usability of the cloth after a few testing sessions. Upon detection of this degradation, the electrodes were replaced throughout testing to ensure the integrity of EEG data. While conductive cloth has been found to be a viable electrode material, as noted by Honiball [45], a custom earpiece design would have been more ideal. However, the logistical challenges of manufacturing 15 custom earpieces for each participant were prohibitive. Despite this limitation, the device successfully captured adequate EEG data and triggered the delivery of the audio stimulus. Future work will focus on enhancing device design for improved user comfort during sleep. Potential upgrades include replacing the Cyton board with a custom EEG board that could integrate data storage and stimulus delivery, potentially eliminating the need for an external laptop. Additionally, alternative electrode materials with better durability and reliability, as well as longer data recording time (up to 8 h), will be considered. Increasing the sample size could yield more statistically significant results, though it remains uncertain if this will guarantee improved outcomes.

The processing laptop, positioned next to the participant, required substantial computational power for continuous data analysis throughout the night, necessitating a constant power supply. Furthermore, this study took place in South Africa during periods of load shedding (long duration of 2–4 h power cuts), which resulted in power interruptions that impacted the duration of some recordings. The unpredictable timing of these outages led to occasional disruptions in the recording process, as the laptop could only remain powered for limited periods. Despite these challenges, the collected data were still deemed valuable for analysis. Given the small sample size, this study was considered a proof of concept, providing a foundation for future research with a larger cohort.

## 5. Conclusions

This study presents the development and initial testing of an in-ear EEG device capable of delivering auditory stimuli and recording EEG signals during sleep. The results suggest that the combined auditory stimulus of ASMR and a 3 Hz binaural beat holds promise for enhancing NREM 3 sleep and promoting delta band activity. While the preliminary results are promising, further testing with a larger sample size is necessary to determine whether these effects can be validated in a broader population. Improvements to the device design, including more durable electrode materials and onboard data processing, will enhance user comfort and ensure reliable data collection in future studies. Overall, this study sets the foundation for continued research into the use of auditory stimuli and portable EEG devices to improve sleep quality and monitor brain activity during sleep.

## Data Availability

The raw data supporting the conclusions of this article will be made available by the authors upon request.
